# 

**Published:** 1898-08

**Authors:** 


### The Bi-State Dental Meeting.

The Bi-State meeting of the Northern Indiana and Southwestern Michigan Dental Societies will be held at Elkhart, Ind., Wednesday and Thursday, September 22n and 23d. All members of the profession are cordially invited to attend.

T. P. ADAMS, *Sec.*

This is one of our live dental Societies. It has been our good fortune to attend its meetings heretofore in one or two instances, and we can bear testimony of the excellence of its work. Its members are thoroughly active in promoting its interest and securing the largest amount of profit from its work. It is very desirable, that there should be a large attendance, not only of the membership, but of visiting members as well.

### To the Omaha Meetings.

The fact of the coming meetings has been quite fully announced through the various journals. The road for those going from Cincinnati and vicinity has not yet been specified. Upon looking the matter up quite fully we can say there is no road more feasible and desirable than the "Missouri Pacific," via St. Louis, Kansas City and on to Omaha. The "Big Four" road from Cincinnati to St. Louis is familiar to all.

The "Missouri Pacific" from St. Louis to Omaha is one of the best roads in the country; the road and its equipments has no superior and its service is admirable.

The better time for those going from Cincinnati and vicinity will be at 8:30 A. M. by "Big Four" or 9 o'clock A. M. by "B. & O. S. W.," arriving at St. Louis in time for the 8 P. M. "Missouri Pacific," and reach Omaha next day at 12.55.

A rate of 1⅓ fare (making about $32 round trip) has been promised, and there is a probability of a still better rate being obtained before the time of going. We trust that Cincinnati and its neighboring towns will be largely represented in the meetings at Omaha.

### Annual Meeting of the National Association of Dental Faculties.

BALTIMORE, Ind., July 18, 1898.

The annual meeting of the National Association of Dental Faculties will be held in the Mercer Hotel, at Omaha, beginning August 26th, at 2 P. M.

It is to be hoped that all members of the Association will be present at that time.

The Executive Committee will meet on the preceding Thursday at 2 P. M.

Colleges are notified to present their business at the first session of the Committee.

By order of JONATHAN TAFT, *Chairman Ex. Com.*
B. HOLLY SMITH, *Sec'y.*

### National Dental Association.

The first annual meeting of the National Dental Association will be held at Omaha, Neb., commencing at 10 A. M. Tuesday, August 30, 1898. GEO. H. CUSHING, *Rec. Sec'y.*

N. B.—I wish to state, owing to the stories in circulation regarding lack of accomodations at Omaha, that I recently visited there to make a personal inspection of the hotels, and found the accomodations ample and rates reasonable.

J. N. CROUSE, *Chairman Ex. Com.*

### CONTENTS.

COMMUNICATIONS.

**A Monthly Magazine Devoted to the Interests of the**

**DENTAL PROFESSION.**

*Terms Reduced to* $2.00 *Per Year. Single Copies,* 25 *Cents.*

**EDITED BY PROF. J. TAFT, D.D.S.**

—AND—

**Published by SAM'L A. CROCKER & CO., Ohio Dental and Surgical Depot.**

The volume commences in January and closes in December.

The Register being issued monthly is always fresh, therefore Indispensable to the practitioner who desires to keep posted up with the new inventions and improvements which are daily developed. Neither expense nor effort will be spared to give value and variety to its contents. Articles will be illustrated with well executed engravings whenever they will add to the interest or assist to explanation.

Its extensive circulation and frequency of issue make it one of the BEST DENTAL ADVERTISING MEDIUMS in the United States.

All subscriptions must begin with January or July.

Local or special notices, five lines or less, will be inserted for $3 each insertion; over five lines, 50 cts. per line.

All advertising bills payable in advance, or at furthest, after the issue of the first number containing the advertisement. All transient advertisements to be paid for in advance.

☞CONTRIBUTIONS SOLICITED.

Exclusively Editorial Communications should be addressed to the Editor.

The latest number of the Register may be ordered with or without other goods [illegible] any time, and all letters should be addre[illegible]

